# Note from the Editors: Striving for a More Reader-Friendly Journal

**Published:** 2005-07

**Authors:** Thomas J. Goehl

**Affiliations:** Editor-in-Chief, *EHP,* National Institute of Environmental Health Sciences, National Institutes of Health, Department of Health and Human Services, Research Triangle Park, North Carolina, E-mail: goehl@niehs.nih.gov

We at *EHP* continually strive to make our content more accessible to our readers. With this issue, we are pleased to unveil some journal format changes that we hope will help with this goal. Our Table of Contents has a new easier-to-read format. Titles are now in boldface, and keyword descriptors have been added. Research Articles for which nonspecialist summaries (Science Selections) have been prepared are identified as such with a small red arrow.

We have also changed the grouping of our peer-reviewed articles. Commentaries & Reviews are now grouped into their own new category. Toxicogenomics articles are being incorporated into the Research section to reflect the rapid acceptance of this critical tool into the environmental health research arsenal. All other peer-reviewed articles are included within an expanded Research section.

As always, the editors welcome your comments as we try to further enhance the value of *EHP* to the environmental health community.

